# Retraction: Frequency and Predictors of Acute Kidney Injury in Patients With Acute Coronary Syndrome in a Tertiary Care Hospital: A Retrospective Study

**DOI:** 10.7759/cureus.r50

**Published:** 2022-03-17

**Authors:** Hemanth Kumar, Daniyal Jilanee, Shivani M Mehta, Amna Gul, Syed Muhammad Huzaifa Shah, Sumaira Saleem, Maria Binte Sarfraz, Syed U Ashraf, Sher Wali

**Affiliations:** 1 Medical School, Maharajah's Institute of Medical Science, Nellimarla, IND; 2 Medical School, Liaquat National Hospital and Medical College, Karachi, PAK; 3 Medical School, Xavier University School of Medicine, Oranjestad, ABW; 4 Medical School, Ziauddin University, Karachi, PAK; 5 Medical School, Dow Medical College, Dow University of Health Sciences, Karachi, PAK; 6 Department of Physiology, Army Medical College, National University of Medical Sciences, Rawalpindi, PAK; 7 Internal Medicine, Dow International Medical College, Dow University of Health Sciences, Karachi, PAK; 8 Research, Indus Hospital & Health Network, Karachi, PAK

This article has been retracted due to the unknown origin of the data, lack of verified IRB approval, and purchased authorships. It was also discovered that the article was not submitted by Sher Wali, but by Rahil Barkat while using the account of Sher Wali. Mr. Barkat was involved in data theft and misuse in two recently published Cureus articles, which have since been retracted.

As the origin of this article’s data and verified IRB approval cannot be confirmed, we have made the decision to retract this article. Cureus has confirmed that the co-authors were asked by Mr. Barkat to proofread the article and provide payment in exchange for authorship. (Proofreading is an insufficient contribution to warrant authorship as defined by ICMJE.) These payments were made in the guise of “editing fees” but greatly exceed any editing fees paid to Cureus. While these authors may have been defrauded by Mr. Barkat, they remain complicit due to their lack of honest contributions to the article.

